# Impact of quality trimming on the efficiency of reads joining and diversity analysis of Illumina paired-end reads in the context of QIIME1 and QIIME2 microbiome analysis frameworks

**DOI:** 10.1186/s12859-019-3187-5

**Published:** 2019-11-15

**Authors:** Attayeb Mohsen, Jonguk Park, Yi-An Chen, Hitoshi Kawashima, Kenji Mizuguchi

**Affiliations:** Artificial Intelligence Center for Health and Biomedical Research (ArCHER), National Institutes of Biomedical Innovation, Health, and Nutrition (NIBIOHN), 7-6-8, Saito-Asagi, Osaka, Ibaraki 567-0085 Japan

**Keywords:** QIIME, Quality trimming, Paired-end reads, Diversity analysis, Optimization

## Abstract

**Background:**

To increase the accuracy of microbiome data analysis, solving the technical limitations of the existing sequencing machines is required. Quality trimming is suggested to reduce the effect of the progressive decrease in sequencing quality with the increased length of the sequenced library. In this study, we examined the effect of the trimming thresholds (0–20 for QIIME1 and 0–30 for QIIME2) on the number of reads that remained after the quality control and chimera removal (the good reads). We also examined the distance of the analysis results to the gold standard using simulated samples.

**Results:**

Quality trimming increased the number of good reads and abundance measurement accuracy in Illumina paired-end reads of the V3-V4 hypervariable region.

**Conclusions:**

Our results suggest that the pre-analysis trimming step should be included before the application of QIIME1 or QIIME2.

## Background

Microbiome studies have attracted much attention recently. Several publications have reported the effects of the microbiome on health and disease, which range from the regulation of metabolism to its relation with diseases, such as the inflammatory bowel disease [1–3]. The composition of human microbiome in the gut, milk, or skin is affected by environmental, dietary, and lifestyle factors. For this reason, microbiome studies are gaining increasing importance, and are not only limited to human and health related issues, but are also being conducted for ecological and environmental purposes [4].

There are several methods to determine the microbial composition of biological samples, microbial culture and nucleotide sequencing being the most important ones. Although microbial culture is the classical method for these kinds of studies, the majority of microbial species cannot be cultured either because we do not have the required biological knowledge to culture them or because more advanced laboratory techniques are needed to create the appropriate culture environment [5]. In other words, the efficiency of the microbial culture method is dependent on the biological characteristics of bacteria, including metabolism and their aerobic or anaerobic nature. In contrast, nucleotide sequencing is neither affected by the biological characteristics of bacteria nor by the currently available technology to mimic the appropriate environment for culturing them. Rather, advanced accurate sequencing technologies are required, which are getting cheaper, faster, and more accurate [6]. Several nucleotide sequencing techniques are currently available, such as the shotgun [7] and 16S rRNA gene sequencing.

Whole genome shotgun sequencing is a new technique that provides more abundant information, especially related to the function, genome content, as well as taxonomic classification. However, it is more expensive and requires complex and computationally-intensive analysis [8].

The amplicon analysis of the 16S rRNA genes is currently the most commonly used method; it has been used in big projects, such as the Human Microbiome Project [9]. The 16S rRNA gene is used because it is highly conserved among microorganisms and contains hypervariable regions that have sufficient variation to allow distinction at individual taxonomic levels. The basic idea is to amplify a selected region of 16S rRNA gene using the polymerase chain reaction (PCR), sequencing the amplified product, and comparing the sequence with a reference database [10].

The accuracy of the results of amplicon analysis of the 16S rRNA genes is dependent on many factors, including the sequenced region, the sequencing technology, and the reference database used for the analysis. There are several strategies to increase the accuracy of the analysis. One strategy is to sequence a longer region of the 16S rRNA gene to obtain enough information for taxonomy assignments. Unfortunately, this task is not free of obstacles. A major problem is the increased error rate along with the position of the sequenced base [11], which is caused by the limitation of the sequencing technologies, and generally is not related to the sample preparation or the steps preceding the sequencing. This problem can be partially solved effectively by using paired-end sequencing technology. In this context, one approach is to sequence a hypervariable region shorter than twice the sequencing ability of the machine to allow enough overlapping regions for the reads to be effectively joined.

The presence of low-quality bases towards the right end of the sequence adversely affects the joining step, leading to the failure of the joining, and consecutively to the loss of the reads in the middle of the analysis. One approach to reduce the consequences of this problem is to trim the reads distal to a point where phred quality score drops below a specific threshold (quality trimming). If the length of the overlapping region is enough for the paired-end reads to be joined effectively, this may lead to reducing the loss of reads because of this process.

In this study, we used QIIME (pronounced as chime [12] and stands for Quantitative Insights into Microbial Ecology), which is a pipeline for microbiome analysis that starts from raw DNA sequencing data and ends with visualization and statistical analysis. It consists of a comprehensive collection of tools [13, 14] that are available at http://qiime.org/. Some of these tools are written in Python by the QIIME developers, while others are incorporated in the pipeline, such as usearch [15] and fastq-join [16, 17], wrapped over by QIIME scripts to work in harmony with the entire pipeline. QIIME is now the standard for microbiome analysis and in particular, for the analysis of Illumina paired-end reads.

Recently, a new version of QIIME, named as QIIME2, was published [14], which is described as “a next-generation microbiome bioinformatics platform that is extensible, free, open source, and community developed”. QIIME2 [18] includes new tools and methods different from the older version, and also provides an application programming interface (API) for automation of the processing and for extending the platform to possible and manageable limits. Even though QIIME1 has been succeeded by the newer version and is not supported anymore by the developer community, who advise investigators to move to QIIME2, it is still extensively used as is evident from several recently published articles.

In this study, we examined the effect of quality trimming prior to the joining of the read pairs on the overall performance of the QIIME pipeline. We tested this effect qualitatively and quantitatively in both the QIIME1 and QIIME2 frameworks.

## Results

We analyzed the results of the full QIIME1 and QIIME2 analyses in both real (RS) and simulated (Sim) samples using the known gold standards. The quality trimming was carried out using bbduk. We passed the –q parameter to that script to create the trimmed samples. Thereafter, we calculated the number of good reads remaining after the quality control steps (“split_library.py” and “identify_chimeric_seqs.py” in QIIME1, and “DADA2 non.chimeric reads” in QIIME2); these two values were used as indicators of the efficiency of analysis. Moreover, we observed that the Sim set samples showed similar results compared to the RS set samples with regard to the above-mentioned parameters. Using the Sim set, we calculated the Euclidean distance of the samples to their gold standard in the principal coordinate analysis (PCoA) space, showing how quality trimming affected the accuracy of determination of bacterial abundance in the samples (Beta diversity).

### The number of good reads

Good reads were those that were merged successfully and passed the quality control and chimera removal steps and were eventually used for the determination of sample composition [operational taxonomic unit (OTU) picking in QIIME1, amplicon sequence variant (ASV) determination in QIIME2, and taxonomy assignment].

In QIIME1, the RS and Sim datasets showed similar behavior of the measurements for the good reads. The increase in the quality trimming threshold lead to an increase in the number of good reads until it reached the maximum at level 12–14, and then decreased.

**Fig. 1:**
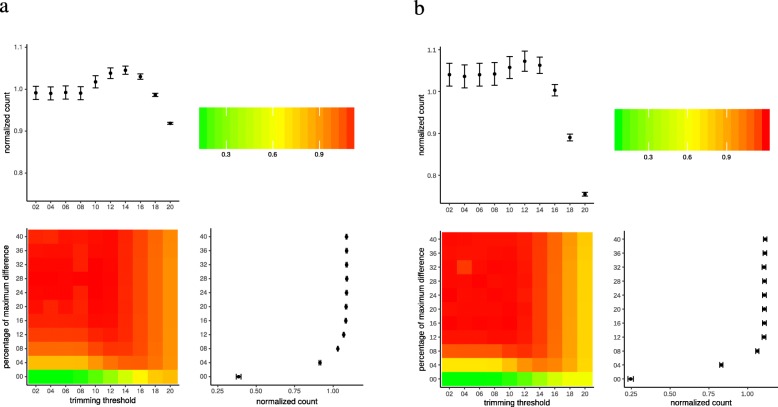
The number of good reads after quality control and chimera removal using QIIME1 in the real sample (RS; **a**) and simulated (Sim; **b**) datasets. Values of the heat map were normalized by dividing each value with the average of the whole data set. Peripheral plots show dots as means, and error-bars represent the standard error of the means

Moreover, the percentage of maximum difference showed a hyperbolic curve. We found that the number of good reads increased substantially from values 0 to 8, and plateaued around 12 (Fig. 1a and b). The heat maps of the normalized good reads for both the RS and Sim datasets showed identical distribution of values suggesting that the Sim dataset was comparable to the RS dataset.

**Fig. 2:**
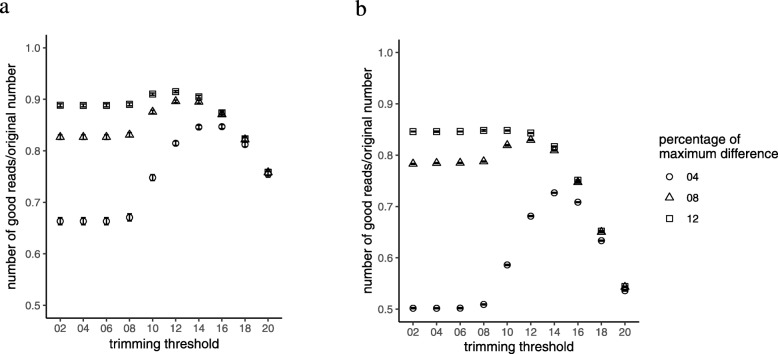
The effect of quality trimming threshold in QIIME1 on the number of good reads divided by the original number of raw reads for each sample in the real sample (RS; **a**) and simulated (Sim, **b**) datasets, with three different values (4, 8, and 12) of percentage of maximum difference. These plots show the means and the error bars represent the standard error of the means

Increased percentage of maximum difference allows more errors to pass to the subsequent steps of the analysis, and therefore, keeping a low percentage of the maximum difference is theoretically better. Accordingly, we chose low values of the percentage of maximum difference for the next steps. We selected the default value (8) for the fastq-join script and two extra values (4 and 12) on the two sides. The RS and Sim sets showed similar results with minimal difference (Fig. 2a and b). As a general rule, using a trimming threshold greater than 14 results in more loss of reads in all the three values of percentages of maximum difference. However, with the default value (8), the trimming significantly affected the number of good reads in both the RS and Sim datasets. This increase reached the maximum at trimming thresholds of 12–14. At the same time, this effect was less prominent for the percentage of maximum difference value of 12, especially in the Sim dataset.

**Fig. 3:**
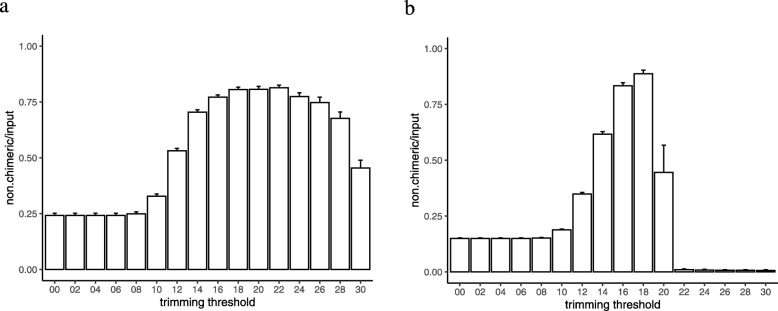
The effect of quality trimming threshold in QIIME2 on the number of good reads (non.chimeric) divided by the number of raw reads (input) for each sample in the real sample (RS; **a**) and simulated (Sim; **b**) datasets. The bar shows the mean, and the error bars show the standard error of the means

The effect of trimming was more prominent on the QIIME2 results. Without trimming, more than 75% of the reads were lost in both the RS and Sim sets (Figs. 3a and b). Trimming at thresholds smaller than 10 did not have any effect; however, this effect was clearly visible at trimming thresholds of 18–22 in the RS set and 18 in the Sim set. In the Sim set, trimming at thresholds greater than 18 resulted in a sudden drop in the number of reads. This drop can be explained by the failure of pair joining because of the quality score profile of the simulated reads.

### Diversity analysis

**Fig. 4:**
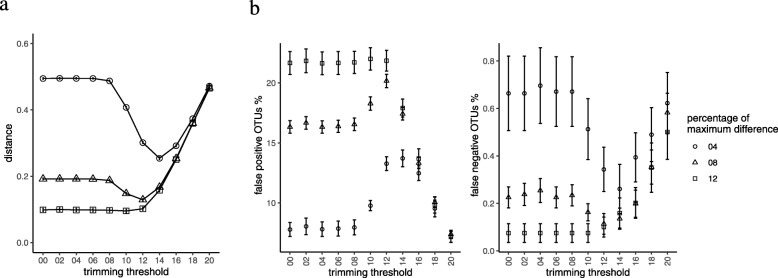
(**a**) The distance of each sample to its gold standard after the principal coordinate analysis using the Jaccard distance for QIIME1 analysis in the simulated (Sim) dataset. (**b**) Operational taxonomic unit (OTU) assignment error: False positive percentage means the percentage of OTUs that were assigned in the samples but were not originally present in the gold standard. False negative percentage is the percentage of OTUs that were not detected in the results but were included in the gold standard

In QIIME1, the distance of the simulated samples (the Sim set) to their gold standard was affected by the quality trimming. With percentage of maximum difference having a value 8 (the default for fastq-join), we could see that the trimming threshold of 12 showed less distance from the gold standard, However, the distance increased significantly with higher trimming thresholds (14 and above). With the percentage of maximum difference having a value 12, the distance was not affected by the trimming (Fig. 4a). Moreover, the errors in the assignment of OTUs showed a complicated behavior (Fig. 4b). Higher values of the percentage of maximum difference lead to more false positive OTU assignments. This was possibly because of the merging of reads with the alignment errors, leading to the wrong OTU assignment. A false negative percentage indicates the percentage of OTUs that was not detected. It was much smaller in comparison to the false positives. From Fig. 4a and b, we can notice that the increase in falsely assigned OTUs did not affect the overall distance among the samples. For example, at a percentage of maximum difference value 12 and a trimming value 12, we could see results that were much closer to the standard although the rate of false positive OTU assignments was higher.

**Fig. 5:**
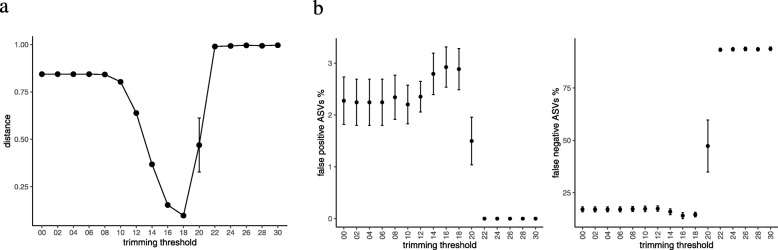
(**a**) The distance of each sample to its gold standard after the principal coordinate analysis using the Jaccard distance for QIIME2 analysis in the simulated (Sim) dataset. (**b**) Amplicon sequence variant (ASV) assignment error: False positive percentage means the percentage of ASVs that were assigned in the samples but were not originally present in the gold standard. False negative percentage is the percentage of ASVs that were not detected in the samples but were included in the gold standard

**Fig. 6:**
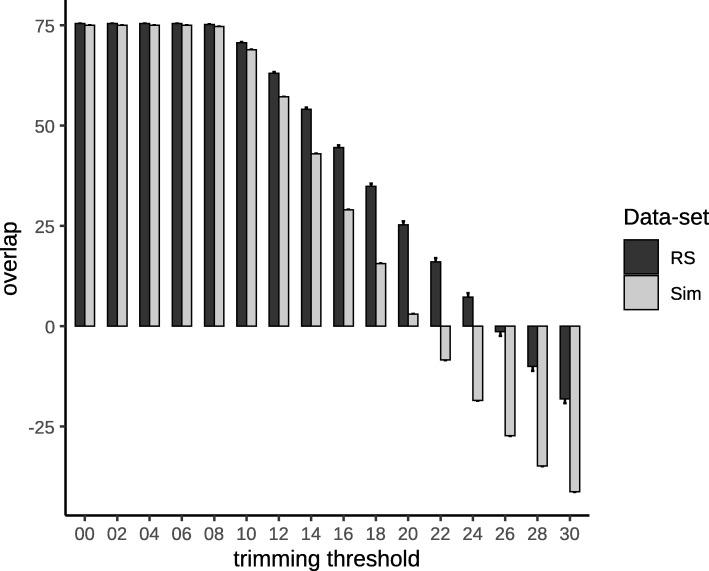
The calculated overlap length between the pair end reads after trimming. RS: Real sample dataset; Sim: Simulated dataset. Negative values represent the calculated gap between the reads, and their merging is impossible

In QIIME2, the distance between the samples and their gold standard started reducing with trimming thresholds of 10–12 and reached its minimum around 18 and increased abruptly, thereafter (Fig. 5a). Moreover, the number of observed ASVs showed minimal but significant increase to reach a maximum around the trimming threshold of 18; it also declined abruptly, thereafter. The reason for this decline was the length of the overlap segment (Fig. 6). In the RS dataset, the overlap was longer, with higher trimming thresholds, compared to that in the Sim set, which can be explained based on the quality profile of these two sets (Additional file 2: Figure S1). In contrast to the results of QIIME1, those of QIIME2 showed higher specificity and lower sensitivity. This can be clearly understood from the results presented in Figs. 4b and 5b. QIIME1 showed high percentage of false positives, which could reach 20% whereas QIIME2 showed only 3% false positives. Moreover, QIIME1 showed a low percentage (< 1%) of false negatives in contrast with > 10% in QIIME2. These results reflect the differences in the quality control strategy between QIIME1 and QIIME2. DADA2, the tool used in our analysis for denoising the data, is more stringent than the quality control steps in QIIME1.

## Discussion

This is the first study that examines the effect of quality trimming thresholds on paired-end Illumina reads in microbiome analysis. However, the effect of quality trimming using multiple tools has been explored in a previous study [19]. Our findings confirmed that quality trimming has a significant effect on the results of microbiome analysis. In QIIME1, with the default value of percentage of maximum difference of fastq-join (8), the quality trimming at thresholds of 10–14 showed the highest number of good reads in both the RS and Sim datasets. It also showed shorter Euclidean distance between the sample and gold standard in the PCoA multidimensional space using the Jaccard distance in the Sim dataset. Although in QIIME2, a trimming threshold of 18 also showed increased number of good reads in both the RS and Sim datasets, it also showed a shorter Euclidean distance between the sample and gold standard in the PCoA space using the Jaccard distance. These values were affected by the overall quality profile of the samples used in the analysis.

Because of the limitations of sequencing technology, the phred quality is reduced as the length of the sequenced region is increased, and therefore, more bases toward the end of reads are likely to be assigned wrongly. As a result, the read-joining is also affected, leading to low quality merged regions, and this might also lead to the discarding of the whole read at the quality control step. Theoretically, discarding the parts with low quality and putative wrongly assigned bases, improves the results of pair-joining, and therefore, of the subsequent analysis steps. In this study, we show this effect in terms of the number of good reads. We also show the effect on the final analysis by demonstrating the change in the alpha diversity of the samples, and the distance of samples to their gold standard in the PCoA space using the Jaccard distance in the simulated data.

Next generation sequencing is used to determine the bacterial abundance in biological samples by assigning the taxonomical information to all the resulted sequences. In many cases, to make the samples comparable, rarefaction is applied, which is the random selection of a specific number of sequences equally in all the samples to be used in subsequent inter-sample comparisons. However, new methods of analysis that do not involve sampling have been suggested [20]. Gaining more reads after merging, quality control, and chimera removal enhance the accuracy of the analysis, especially for bacterial strains with low population in the sample, which might not be detectable. Therefore, we show that the number of reads were increased in the RS data, which means increased accuracy of the result. We also show, using the Sim dataset, that the distance of each sample to its gold standard was similarly decreased. These findings imply that, by applying quality trimming, we can perform better abundance analysis.

Even though the results of this study are limited to the V3-V4 hypervariable regions sequenced using Illumina paired-end sequencing technology, this technology is extensively employed in microbiome research. It is one of the most commonly used technology for DNA sequencing, especially in microbiome analysis; we searched the Sequence Read Archive (SRA) repository of NCBI [21] using the query, “metagenomic”[source] AND “illumina”[Platform] AND “paired”[Layout], and it returned more than 680,000 hits, representing more than half (56%) of the total metagenomic entries. Moreover, most of the studies on the microbiome target either the V3 or V4 region, or a combination of two or more regions. In case, a combination of two regions are sequenced, the most common combination is that of V3 and V4 [10].

Compared to QIIME1, which applies quality check steps after merging the paired-ended reads, QIIME2 applies quality check steps before merging, when the DADA2 [22] plugin is used. DADA2 uses a different strategy based on ASV rather than clustering the OTUs used in the QIIME1 uclust algorithm. This new method is suggested to be more accurate and comprehensive [23]. Our results show that using DADA2 makes the method more specific and less sensitive than QIIME1; however, further investigations are required to support this notion.

We also did the same analysis using mock community samples (data not shown); however, when we calculated the distance of the samples to the gold standard using principal coordinate analysis, we failed to see useful results because the gold standard was much far from all the samples. That was possibly caused because of the effect of multiple steps of processing, starting with community preparation and ending with QIIME analysis, passing thorough amplification and sequencing. The result can also be affected by the reference database used [24]. To overcome these obstacles, we generated simulated data samples to help us accomplish our goal to understand the effect of quality trimming alone, without allowing other interfering factors to affect the results. For this, we wrote a novel simple script that produces two sets of samples—one set with real quality scores derived from real samples and another set with no introduced errors and high quality scores (40) to be used as a gold standard. The use of a gold standard sample helped us investigate the effect of quality trimming without being affected by the reference database or analysis pipeline used.

To make the simulated data diverse and more generalized, we used the greengenes database as a reference to generate the samples, and SILVA to analyze them. We also used phred quality scores different from those for the real dataset. We collected a large number of quality scores from different kinds of samples. This resulted in different optimum trimming thresholds in the Sim data compared to that in the RS data. For example, trimming at a threshold of 20 gave good results for the RS data, whereas it resulted in poor results for the Sim data, the reason for which was related to the difference in the overlapping region. In the Sim data, the average number of overlapping nucleotides was less than 10 (Fig. 6), which is not enough to allow merging of the paired end reads, whereas in the RS data, the average number of overlapping nucleotides was more than 20. For further elucidation, the quality plots of a sample from both the datasets are shown in Additional file 2: Figure S1.

Our results also suggest the importance of the number of overlapping nucleotides. This number can help in accurately deciding the trimming threshold for the used dataset by applying our protocol to the dataset and plotting the overlapping threshold. That should be less computationally expensive than conducting the full analysis several times with different parameters. Moreover, our method can also be applied by generating one or few samples using the quality profiles of the dataset under analysis, and applying the full analysis and comparing the results with the gold standard. This method is obviously promising; however, more testing and evaluation might be needed.

We suggest other parameters, such as the sequenced region and sequenced length, to be investigated in future studies. We also suggest that our protocol be followed to obtain further insights using different datasets. Conducting such analyses needs a significant programming effort and would be computationally expensive, especially with QIIME1. However, we have created Auto-q script (Additional file 1) [25], which helped us automate the entire process in QIIME1. We also suggest that our approach can be used in the future to create a tools to decide the optimal parameters such as machine learning models.

## Conclusions

Quality trimming at an appropriate quality threshold leads to an increase in the number of good reads that pass quality control steps in both QIIME1 and QIIME2 pipelines. Based on our results, we recommend trimming thresholds of 10–14 for QIIME1 and 18 for QIIME2 for the V3-V4 hypervariable regions sequenced using Illumina paired-end reads sequencing technology; however these values can be affected by the quality profile of the samples.

## Methods

### Sample data

The real samples (RS) set consisted of 30 sequences of human fecal samples, downloaded from the SRA database [21]. These samples were sequenced using Illumina paired-end technology, with a length of 300 bases for each read pair. We chose paired-end reads from Illumina because it is the most commonly used technology, and chose 300 bases for the V3-V4 region because it provides enough overlap length to allow trimming to be done without losing reads in the merging process. We randomly selected the samples of the V3-V4 hypervariable region, with accession codes SRR5446821–SRR5446850.

Sim set: Simulated data set consisted of 10 samples. The Sim set was generated using an in-house script. It was built on the greengenes database with 97 similarities “gg_13_8_otus/rep_set/97_otus.fasta” using S-D-Bact-0341-b-S-17 and S-D-Bact-0785-a-A-21 primers [26]. For each sample a gold standard was generated with no quality errors, following the steps mentioned below:

1. The reference sequence used to generate the simulated data was: “gg_13_8_otus/rep_set/97_otus.fasta” from the greengenes database.
2. Extraction of the V3-V4 region: The reads in the reference file were truncated using the S-D-Bact-0341-b-S-17 and S-D-Bact-0785-a-A-21 primers. To detect the primer position, we used the “nt_search” function from the “BioPython SeqUtils” package [27]. The extracted and truncated reads represented the V3-V4 hypervariable region, saved in the V3-V4.fasta file.
3. Generation of paired-end reference files: The V3-V4 sequences were truncated at length 310 from both the sides. The reverse side read was reversed using the “reverse_complement” method in the BioPython package. This step resulted in two fasta files (“ref_R1.fasta” and “ref_R2.fasta”).
4. Extraction of quality scores: we extracted the quality scores from multiple R1 and R2 fastq files retrieved from multiple publicly available datasets of the V3-V4 region. The quality scores were randomly ordered and saved in two separate text files. All the sequence data for these samples were discarded.
5. Generation of sample fastq file pairs: A random number of sequences was selected from “ref_R1.fasta” and “ref_R2.fasta”. The number of copies for each sequence was randomly assigned and a set of quality sequences was also randomly assigned to each copy using the pool of quality scores. The nucleotides in each read were manipulated depending on the probability of the incorrect base call using the choice function in the numpy random package in python [28]. Using this function, we can assign probabilities, using the quality scores, for the possible choices. For instance, if the correct nucleotide is “C” and the quality score is 2, it means the probability of the correct answer is 0.63. We give “C” a probability of 0.63 whereas the other three nucleotides (“G”, “A”, and “T”) are given probabilities of 0.123 each, which is equal to (1–0.63)/3.
6. Generation of gold standard: for each sample, another fastq files pair was generated using the same reference sequences without manipulating the nucleotides. All the quality scores in these two files were set at 40. These two files were used as the gold standard for that sample.

### Primer and quality trimming

We trimmed the first 17 bases from the forward reads and the first 21 bases from the reverse reads. Thereafter, we applied quality trimming, which is the process of trimming the right side of the read when the quality of the base is reduced below a certain threshold. We used BBDuk from bbtools using BBDuk version: 37.22 [29]. BBDuk is designed to decontaminate the reads using k-mers. (Duk: decontamination using k-mers); however, we used it only for quality trimming. We passed r (right side trimming) for “-qtrim = r”, and passed the even values from 0 to 20 for QIIME1 and 0 to 30 for QIIME2, inclusive of quality trimming thresholds to “-trimq” parameter.

### QIIME analysis

For the QIIME1 (Version 1.9.1) analysis, we followed the steps mentioned below:

1. Joining of the read pairs: we used fastq-join for merging the paired reads. Fastq-join allows users to decide the percentage of maximum difference, which means the percentage of bases with a different assignment. Increasing the percentage of maximum difference means allowing more errors to pass to the next step in the analysis process. We did the pair joining for all values of percentage of maximum difference from 0 to 40 with step 4 including 0 and 40. The joined read quality was assigned using the highest value, if the base was assigned similarly in both the forward and reverse reads. If there was a mismatch, the base with the highest quality value was assigned and given a quality value equal to the difference of the quality of both the bases.
2. Quality control is the step where the reads after joining of pairs with average quality less than a certain threshold is discarded. We used split_library.py script with default parameters from QIIME1.
3. Chimera removal using usearch version 6.1 wrapped by identify_chimeric_seqs.py script from QIIME1. We used the SILVA [30, 31] database (version 128) with 97% similarity as a reference for this step.
4. For OTU picking, we used the pick_otus.py script, with closed reference option. We used the SILVA database (version 128) with 97% similarity.

For QIIME2 analysis, we imported the trimmed fastq files resulting from “primer and quality trimming” step to the QIIME2 artifact. Thereafter, we applied DADA2 to denoise, joined the paired reads, and removed the chimera. DADA2 [32] is originally an R package that is wrapped by the QIIME2 plugin. We applied the default parameter with truncation length for both (forward and reverse) reads as zero.

### Diversity and statistical analysis

The resulted data were exported as a BIOM table and imported to R. The diversity analysis was done using the Phyloseq [20] package from R. The principal component analysis was done using the ade4 package [33] from R with Jaccard distance from the vegan R package [34]. The sample-gold standard Euclidean distance for each sample representation in the PCoA space and its corresponding gold standard was measured. Other statistical analyses and plotting were done in R (ggplot2 [35]) and Python (Pandas [36] and matplotlib [37] packages).

## Supplementary information

**Additional file 1: ** Auto-q automation script details.

**Additional file 2: Figure S1.** The quantiles of phred quality scores for a random sample from both RS and Sim sets.

## Data Availability

RS data are available at SRA repository: SRR5446821–50 [38], Simulated data and the code used to generate the simulated data and the analysis code is available from: https://github.com/attayeb/quality_trimming Auto-q code is available from: https://github.com/attayeb/auto-q

## Abbreviations

API: Application Programming Interface
ASV: Amplicon Sequence Variant
DADA: Divisive Amplicon Denoising Algorithm
OTU: Operational Taxonomic Unit
PCoA: Principal Coordinate Analysis
QIIME: Quantitative insights into microbial ecology
