# Supplementary material for: Impact of quality trimming on the efficiency of reads joining and diversity analysis of Illumina paired-end reads in the context of QIIME1 and QIIME2 microbiome analysis frameworks
Source: BMC Bioinformatics. 2019 Nov 15;20:581. doi: 10.1186/s12859-019-3187-5 (PMC6858638; doi:10.1186/s12859-019-3187-5)
Supplement: Supplementary file 1 — Additional file 1: Auto-q automation script details. [file 12859_2019_3187_MOESM1_ESM.pdf]

Auto-q: Qiime Analysis automating script

Background

Microbiome studies have attracted much attention recently and many publications have reported the effects of the microbiome on health and disease. With the progress of next-generation sequencing techniques and the increased volume of data produced during these studies, the necessity of the automation of the sequencing analysis is increasing day by day.

QIIME (pronounced as chime) stands for Quantitative Insights Into Microbial Ecology. It is a pipeline for microbiome analysis, starting from raw DNA sequencing data and ending with visualization and statistical analysis results. It consists of a comprehensive collection of tools, available at http://qiime.org/. Some of these tools are newly written in Python by the QIIME developers, and other tools are adopted from external sources, such as usearch and fastq-join (https://github.com/ExpressionAnalysis/ea-utils), wrapped over by QIIME scripts to work in harmony with the whole pipeline.

Since QIIME is command line-based, a good experience in terminal and shell scripting is required to perform the multi-step analysis, especially with multiple files, which is usually the case in microbiome analysis. Each step needs to be executed separately. Moreover, in trimming, merging, and chimera removal, it needs to be executed for each single sample. These issues make the analysis strenuous and

prone to mistakes, particularly if the analysis needs to be repeated more than once, for purposes of comparing the effect of analysis parameters, for instance, or to use different reference databases.

When our group started a new microbiome project, we needed to repeat the analysis several times to explore the impact of different data processing methods and parameters, and to decide the most appropriate set of parameters for our data set. Doing such an analysis using the usual QIIME commands is time-consuming and difficult to be tracked, because of the large number of commands to be executed and modified each time. Moreover, we needed to stop the analysis at certain steps, check the intermediate results, and resume the execution, rather than running the entire sequence of commands at once. These two factors motivated us to write a new tool to help achieve these tasks.

We wrote a Python script, Auto-q, to automate QIIME analysis of Illumina paired-end reads. The command-line input is kept to minimal; only one command needs to be executed to perform the full analysis. The raw sequencing data need to be saved as FASTQ files in a single folder. The script takes the input and the output folder names as command-line arguments; all the results and intermediate files are stored in the output folder and its subfolders. Figure 1 shows the sequence of steps followed:

1. Trimming: Illumina per-base sequence quality decreases with the read length, especially in the reverse reads. Therefore, to improve the merging quality, trimming is used to cut the 3' end; the trimming point depends on the quality of the read, and trimming can lead to improved merging results. Since QIIME does not provide any trimming tools, Auto-q wraps around BBDuK from BBTools (http://jgi.doe.gov/data-and-tools/bbtools/). Contaminants are also removed in this step, such as adapter and Phix sequences.

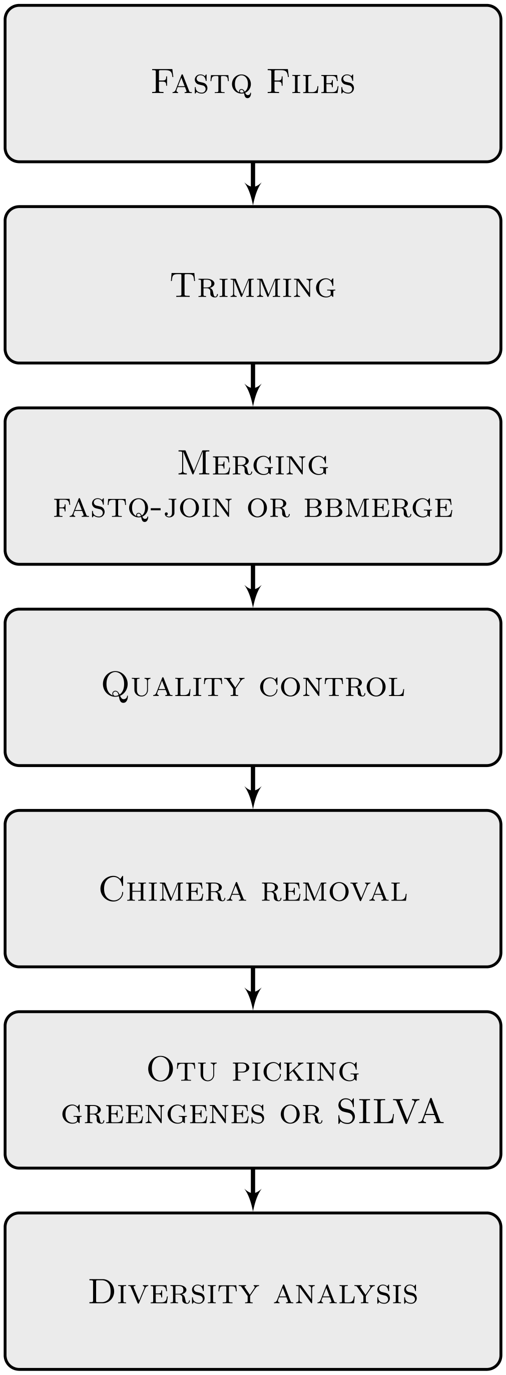

Figure. 1. Auto-q workflow steps.

2. Paired reads joining: The default joining tool in QIIME is fastq-join, and we set it as default in Auto-q as well. We also incorporated BBMerge from BBTools, which offers more options and parameters. After merging, reads shorter than a user-specified cutoff are discarded.

3. Quality control: Using QIIME split_libraries_fastq.py script, Auto-q is able to demultiplex the reads, but in this case, we use it for quality control only. The low-quality reads are removed totally. The quality phred threshold can be specified using the –q argument (19 by default).

4. Chimera removal: It is achieved by applying usearch61 in two steps: first by identifying chimeric sequences using the reference database with the identify_chimeric_seqs.py script from Qiime and then, by filtering the chimera using the filter_fasta.py script.

5. OTU picking: It is performed using the open_reference_otus.py script. The default reference database is Greengenes. We also included SILVA.

6. Diversity analyses: Alpha and beta diversity analyses are performed using the core_diversity_analyses.py script from QIIME.

Other command-line arguments include: -e for the depth of rarefaction for diversity analysis, -s (stop at) to stop the analysis at a particular step, -b (begin with) to restart the analysis (e.g., starting with OTU picking or diversity analysis), and -c for specifying the configuration file.

Auto-q uses a configuration file, a Python INI file, to save the required information such as the location of the database folders, the number of parallel jobs to be executed at the same time, and the default names of the output folders. Mapping file preparation: Auto-q generates a minimally required QIIME mapping file automatically. This file needs to be modified manually to add experimental conditions, and

Auto-q can rerun the diversity analysis. We recommend running the full analysis once, and repeating the diversity analysis as many times as required depending on the experimental conditions being considered.

Auto-q is designed to work in QIIME virtual machine. It is written in Python and utilizes the tools that are already included in QIIME virtual machine, except for a few additions, namely BBTools, and reference databases (SILVA); the installation of these additional tools is trivial without dependency concerns.

References

1. Bushnell B, Rood J, Singer E. BBMerge – Accurate paired shotgun read merging via overlap. PLOS ONE. 2017;12:e0185056.

2. Caporaso JG, Kuczynski J, Stombaugh J, Bittinger K, Bushman FD, Costello EK, et al. QIIME allows analysis of high-throughput community sequencing data. Nat. Methods. 2010;7:335–6.

3. DeSantis TZ, Hugenholtz P, Larsen N, Rojas M, Brodie EL, Keller K, et al. Greengenes, a Chimera-Checked 16S rRNA Gene Database and Workbench Compatible with ARB. Appl. Environ. Microbiol. 2006;72:5069–72.

4. Edgar RC. Search and clustering orders of magnitude faster than BLAST. Bioinformatics. 2010;26:2460–1.

5. Kõljalg U, Nilsson RH, Abarenkov K, Tedersoo L, Taylor AFS, Bahram M, et al. Towards a unified paradigm for sequence-based identification of fungi. Mol. Ecol. 2013;22:5271–7.

6. Quast C, Pruesse E, Yilmaz P, Gerken J, Schweer T, Yarza P, et al. The SILVA

ribosomal RNA gene database project: improved data processing and web-based tools. Nucleic Acids Res. 2013;41:D590–6.

7. Shreiner AB, Kao JY, Young VB. The gut microbiome in health and in disease. Curr. Opin. Gastroenterol. 2015;31:69–75.
