# Supplementary material for: Impact of quality trimming on the efficiency of reads joining and diversity analysis of Illumina paired-end reads in the context of QIIME1 and QIIME2 microbiome analysis frameworks
Source: BMC Bioinformatics. 2019 Nov 15;20:581. doi: 10.1186/s12859-019-3187-5 (PMC6858638; doi:10.1186/s12859-019-3187-5)
Supplement: Supplementary file 2 — Additional file 2: Figure S1. The quantiles of phred quality scores for a random sample from both RS and Sim sets. [file 12859_2019_3187_MOESM2_ESM.pdf]

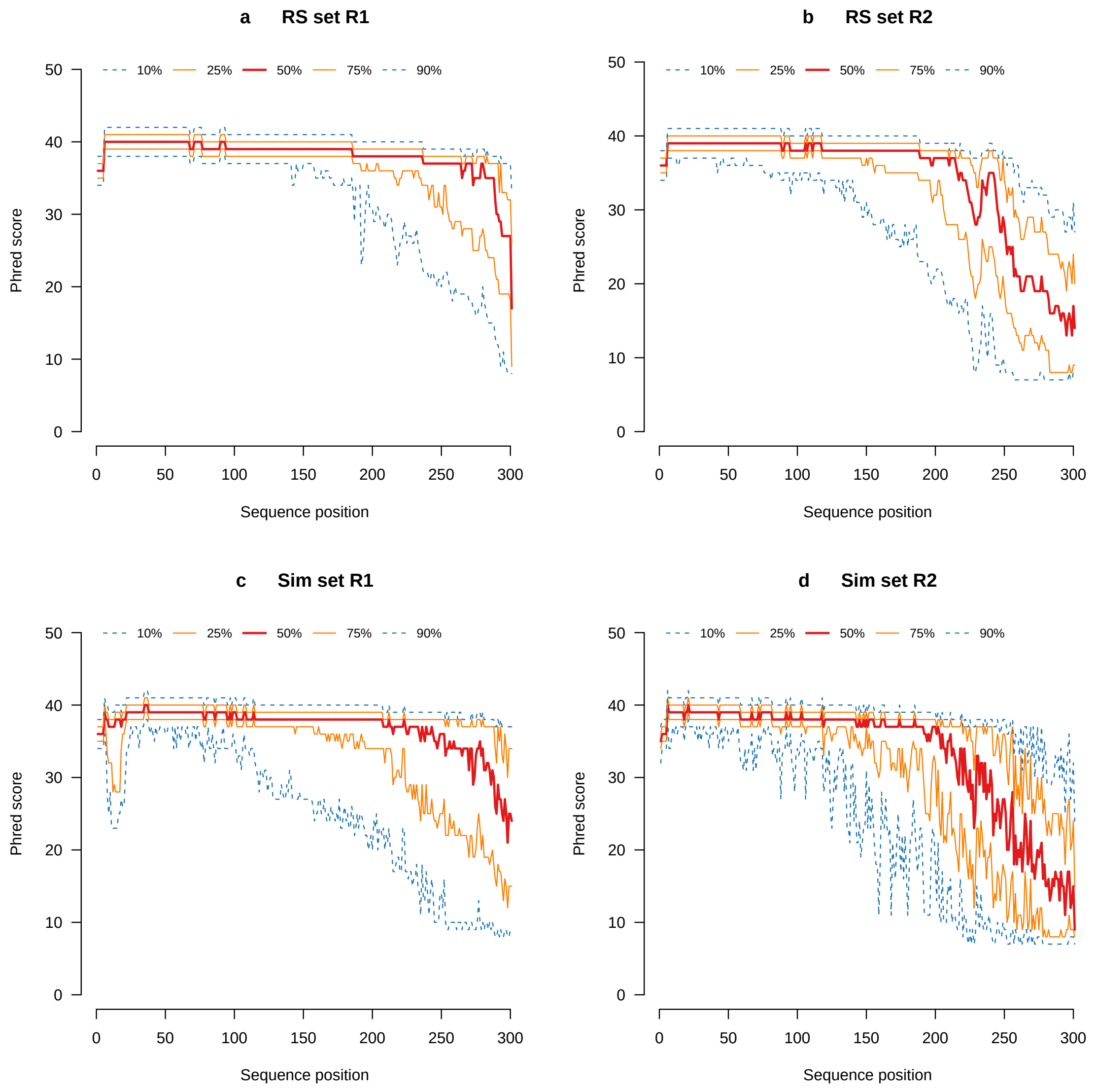


**Supplemenary figure 1:** The quantiles of phred quality scores for a random sample from both RS and Sim sets. Sim set samples show lower quality scores compared to RS set.
